# CRISPR/Cas9-induced structural variations expand in T lymphocytes *in vivo*

**DOI:** 10.1093/nar/gkac887

**Published:** 2022-10-16

**Authors:** Jinchun Wu, Ziye Zou, Yang Liu, Xuhao Liu, Zhengrong Zhangding, Mo Xu, Jiazhi Hu

**Affiliations:** The MOE Key Laboratory of Cell Proliferation and Differentiation, School of Life Sciences, Center for Life Sciences, Genome Editing Research Center, Peking University, Beijing 100871, China; National Institute of Biological Sciences, Zhongguancun Life Science Park, Beijing 102206, China; Tsinghua Institute of Multidisciplinary Biomedical Research, Tsinghua University, Beijing 102206, China; The MOE Key Laboratory of Cell Proliferation and Differentiation, School of Life Sciences, Center for Life Sciences, Genome Editing Research Center, Peking University, Beijing 100871, China; The MOE Key Laboratory of Cell Proliferation and Differentiation, School of Life Sciences, Center for Life Sciences, Genome Editing Research Center, Peking University, Beijing 100871, China; The MOE Key Laboratory of Cell Proliferation and Differentiation, School of Life Sciences, Center for Life Sciences, Genome Editing Research Center, Peking University, Beijing 100871, China; National Institute of Biological Sciences, Zhongguancun Life Science Park, Beijing 102206, China; The MOE Key Laboratory of Cell Proliferation and Differentiation, School of Life Sciences, Center for Life Sciences, Genome Editing Research Center, Peking University, Beijing 100871, China

## Abstract

CRISPR/Cas9 has been adapted to disrupt endogenous genes in adoptive T-lymphocyte therapy to prevent graft-versus-host disease. However, genome editing also generates prevalent deleterious structural variations (SVs), including chromosomal translocations and large deletions, raising safety concerns about reinfused T cells. Here, we dynamically monitored the progression of SVs in a mouse model of T-cell receptor (TCR)-transgenic T-cell adoptive transfer, mimicking TCR T therapeutics. Remarkably, CRISPR/Cas9-induced SVs persist and undergo clonal expansion *in vivo* after three weeks or even two months, evidenced by high enrichment and low junctional diversity of identified SVs post infusion. Specifically, we detected 128 expanded translocations, with 20 615 as the highest number of amplicons. The identified SVs are stochastically selected among different individuals and show an inconspicuous locus preference. Similar to SVs, viral DNA integrations are routinely detected in edited T cells and also undergo clonal expansion. The persistent SVs and viral DNA integrations in the infused T cells may constantly threaten genome integrity, drawing immediate attention to the safety of CRISPR/Cas9-engineered T cells mediated immunotherapy.

## INTRODUCTION

T-cell receptor (TCR) and chimeric antigen receptor (CAR) T-cell-mediated immunotherapy has been successfully applied to treat cancers (1–5). For conventional TCR or CAR T cells, T lymphocytes are isolated, activated, and transfected with TCR- or CAR-expressing vectors *ex vivo*. After expansion, engineered T cells are purified and then infused into the patient to target the tumor cells (4,6). The infused T cells can persist for years and undergo clonal expansion in patients (7–9). Recently, universal TCR or CAR T cells, termed allogeneic T cells, were generated by simultaneously disrupting endogenous TCRs, beta-2 microglobulin (B2M) and programmed cell death protein 1 (PD-1) *via* CRISPR/Cas9-mediated genome editing (4,10–12), which may greatly extend the application of adoptive T-cell therapy.

CRISPR/Cas9-mediated genome editing frequently induces structural variations (SVs), including chromosomal translocations, large deletions, and vector insertions (13–15). SVs greatly threaten the safety of gene editing in clinical applications, as both lymphomas and leukemia are mainly caused by chromosomal translocations (16,17). Genome-wide chromosomal translocations occur at a frequency of 0.1–1% at dozens of target loci in CRISPR/Cas9-edited cells (13,14,18,19). Since billions of edited TCR or CAR T cells are infused into a patient, millions of TCR or CAR T cells with chromosomal translocations are transferred into the patient in each treatment. In this context, chromosomal translocations have been occasionally reported in preclinical human T cells engineered with gene-editing tools, such as transcription activator-like effector nucleases (TALENs) or CRISPR/Cas9 (4,20–22). Recently, chromosomal translocations in engineered T cells caused the FDA to cancel a clinical trial with allogeneic CAR T cells (21).

Chromosomal translocation requires the fusion of two DNA double-stranded breaks (DSBs), either from DSBs at on-target or off-target sites or from genome-wide DSBs induced by cellular stresses or physiological activities (23). Previous studies have reported occasional translocations between two on-target sites in human T cells months post treatment (4). Additionally, chromosomal translocations between on-target site and genome-wide DSBs have also been found in mouse embryonic stem cells 2 weeks post-editing *in vitro* (20). However, the persistence and propagation of genome-wide chromosomal translocations has not been comprehensively profiled *in vivo*. In addition, both large deletions and vector integrations are prevalent during gene editing *in vitro* (13,24) and *in vivo* (25,26), which is another potential cause of pathogenesis (15). Although large structural variants at on-target and off-target site caused by CRISPR/Cas9 can be observed in the offspring of edited zebrafish (27), the progression of SVs and vector insertions in edited animals remains elusive.

To address these remaining issues, we comprehensively profiled CRISPR/Cas9-induced SVs in engineered T lymphocytes from a mouse model of TCR-transgenic (TCR-Tg) T-cell adoptive transfer, which mimics TCR or partially CAR T therapeutics. CRISPR/Cas9-edited T cells from *Helicobacter hepaticus-* (*H. hepaticus-*) specific TCR-Tg mice (*HH7-2tg*) were infused into *Rag1^–/–^* mice to elicit T cell-induced intestinal inflammation for three weeks or up to two months. The levels of CRISPR/Cas9-induced small insertions and deletions (indels) remained constant regardless of infusion. Levels of SVs, especially translocations and large deletions, were comparable before and after infusion for three weeks or even two months. Moreover, dozens of translocations, large deletions and viral DNA integrations were stochastically amplified, with up to thousands of copies, accompanied by T-cell clonal expansion.

## MATERIALS AND METHODS

### T-cell activation, genome editing and infusion

T cells were cultured in RPMI-1640 medium supplemented with 10% fetal bovine serum (FBS), 10 mM HEPES, 100 mM nonessential amino acids (NEAAs), 50 U/ml penicillin, 50 mg/ml streptomycin, and 50 mM β-mercaptoethanol unless otherwise indicated. For the retroviral vector (RV) experiment, TCR transgenic naïve CD4^+^ T cells were sorted from an HH7-2tg Cas9 mouse and then activated under T_H_0 conditions in plates coated with α-mouse CD3ϵ (BioXcell) and α-mouse CD28 (BioXcell) for 2 days. The medium was supplemented with 1 μg/ml α-mouse IFNγ (BioXcell), 1 μg/ml α-mouse IL-4 (BioXcell), and 20 U/ml hIL-2 (Peprotech) to prevent T_H_1 and T_H_2 polarization. Activated T cells were transduced with pST-sgRNA RV (sgRNA target sequence: 5’-GCGGTGAGTCGTGATCTGAG-3’) in the presence of 8 mg/ml polybrene during spin infection (2000 × g for 120 min at 33°C) and then rested for 3 days in medium containing 20 U/ml hIL-2 (Peprotech), 5 ng/ml mouse IL-7 (Sinobio), 1 μg/ml α-mouse IFNγ (XMG1.2, BioXcell) and 1 μg/ml α-mouse IL-4 (11B11, BioXcell). CD90.1-positive activated T cells were sorted and transferred into *H. hepaticus* colonized *Rag1^–/–^* recipient mice, with 300,000 cells per recipient i.v. through the tail vein. After 21 days or 2 months, recipient mice were sacrificed and dissected to obtain intestinal tissues. The intestinal tissues were washed with PBS with 1 mM DTT for 10 min, followed by 5 mM EDTA for 20 min to remove epithelial cells, and then treated with RPMI containing collagenase D (1 mg/ml collagenase; Roche), DNase I (100 μg/ml; Sigma), dispase (0.05 U/ml; Worthington) and 10% FBS at 37°C for 60 min with constant mixing. Leukocytes were collected via a 40–80% Percoll gradient. Inflammatory T cells were sorted as CD3^+^CD4^+^CD45.1^+^CD90.1^+^ for PEM-seq analysis. Antibodies were purchased from BioLegend and eBiosciences: CD3 (145–2C11), CD4 (GK1.5), CD45.1 (A20) and CD90.1 (OX-7). Mice were kept and cultured following the standards of the animal facility in the National Institute of Biological Sciences. Animal experiments were permitted by committee at the National Institute of Biological Sciences.

### Flow cytometry analysis for inflammation

T cells were isolated from the *H. hepaticus*-colonized *Rag1^–/–^* recipient mice after a 3-week infusion, incubated in RPMI with 10% FBS, 50 ng/ml phorbol 12-myristate 13-acetate (PMA, Sigma), 1 μg/ml ionomycin (Sigma), and GolgiStop (BD) for 4 hours, and then stained for CD4 (RM4-5). After that, cells were fixed, permeabilized, and subjected to IFNγ (XMG1.2, BioXcell) staining. Flow cytometry data were acquired on a BD LSRFortessa Cell Analyzer. IFNγ-positive cells among CD4-positive cells were gated and analyzed with FlowJo 10.4.

### PEM-seq and editing outcome identification

PEM-seq was performed as described previously (14,28). Briefly, 20 μg of genomic DNA from naïve, activated, or inflammatory T cells was fragmented to 300 bp in size by sonication. DNA fragments were subjected to *Bst* polymerase 3.0 (NEB)-mediated one-round linear primer extension with a biotinylated primer targeting the on-target site in the first intron of *c-Myc*. After that, the excess biotinylated primers were removed with AxyPrep Mag PCR Clean-Up beads (Axygen). DNA was denatured at 95°C for 5 min, immediately chilled on ice for 3 min, and incubated with Dynabeads MyOne Streptavidin C1 (Thermo Fisher) for 2–4 h. The C1 beads were thoroughly washed with 1× B&W buffer (5 mM Tris–HCl, pH 7.4; 1 M NaCl; 1 mM EDTA), 10 mM NaOH, and 10 mM Tris–HCl and then ligated with a bridge adapter containing random molecular barcodes. After overnight incubation, the ligated DNA on C1 beads was thoroughly washed with 1× B&W buffer and 10 mM Tris–HCl and tagged with Illumina adapter sequences. DNA ranging from 300 to 700 bp was recovered and sequenced on a HiSeq platform (2 × 150 bp).

Sequencing reads were processed by the ‘PEM-Q’ pipeline with the default setting (28). Particularly, a genome containing mm10 assembly of the mouse genome and the viral genome was used for the alignment of sequencing reads. Perfect rejoining means the reads contain the same sequence as the reference genome around the on-target site. Small or large deletions are located in the ≤100 bp or 0.1–500 kb regions from the on-target site, respectively. Junctions are the other events between the on-target site and genome-wide or exogenous DNA. Of note, translocations are the junctions between the on-target site and regions in another chromosome or outside of ±500 kb of the on-target site. For hotspot identification, translocation junctions were normalized to the same editing events, and peaks were called by MACS2 *bdgpeakcall* with the parameter of *-l 1 -c 5*.

## RESULTS

### The main editing outcomes in the engineered T cells remain constant *in vivo*

**Figure 1.**
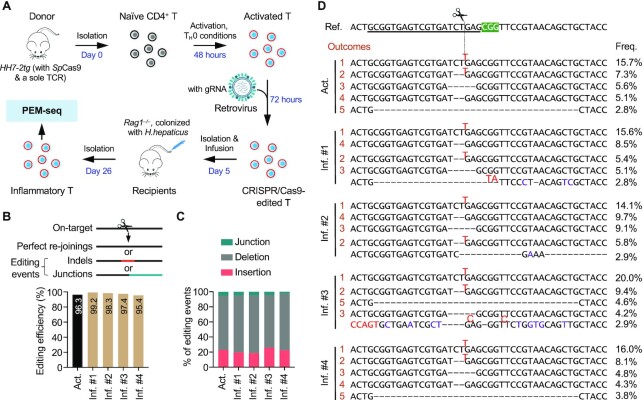
Global analysis of CRISPR/Cas9-induced editing products in T cells before and after infusion. (**A**) Experimental procedures for tracing the *in vivo* progression of gene editing outcomes in engineered T cells. Naïve T cells were isolated from an *HH7-2tg* mouse (male, 12 weeks old, expressing *Sp*Cas9 and a transgenic TCR, HH7-2) by FACS and then incubated with α-CD3/CD28, α-IFNγ, and α-IL-4 for 2 days *in vitro*. After that, retroviruses harboring guide RNA (gRNA), targeting the first intron of *c-Myc*, were introduced into the activated T cells for 3 days. Cells expressing gRNA were sorted out for PEM-seq analysis, and 3 × 10^5^ sorted cells were infused into each of four *Rag1^–/–^* mice (male, 12 weeks old) that were colonized with *H. hepaticus*. Three weeks after infusion, the engineered donor-derived inflammatory T cells in the large intestine were sorted for PEM-seq analysis to comprehensively quantify each editing outcome. (**B**) Top: Schematics of editing outcomes detected by PEM-seq. Bottom: Bar plot showing the editing efficiency of CRISPR/Cas9 in the indicated samples by computing the percentage of editing events among total events. Act., activated T cells; Inf., inflammatory T cells from four littermates 3 weeks after infusion (#1 – #4); indels, insertions, and deletions. (**C**) Proportions of junctions, deletions, and insertions among the editing events. (**D**) The top five products in the activated (orange numbers) or inflammatory T cells at the on-target site. The gRNA sequence is underlined in the reference sequence (ref.). The scissor and green shadow indicate the breakpoint and protospacer adjacent motif (PAM) of *Sp*Cas9, respectively. The top 5 outcomes in activated T cells are labeled with orange numbers, and the occurrence of the same products in inflammatory T cells is indicated with the same number. Insertions (red), mutations (purple), and deletions (‘–’) are indicated in each product. Frequencies (freq.) of each product in the indicated samples are listed on the right.

To trace the behavior of SVs in gene-edited T cells *in vivo*, we employed a mouse model of inducible inflammation in the large intestine (29). Naïve T cells harboring an *H. hepaticus*-specific transgenic TCR, HH7-2tg, were isolated by fluorescence-activated cell sorting (FACS) from an *HH7-2tg* male mouse, 12 weeks old, which also constitutively expressed *Streptococcus pyogenes* Cas9 (*Sp*Cas9) (Figs. 1A and Supplementary Table S1). Isolated naïve HH7-2tg T cells were activated in plates coated with α-CD3/CD28 under T_H_0 conditions *in vitro*. During activation, proliferating T cells were transfected with retrovirus harboring a single guide (sg)-RNA targeting the first intron of *c-Myc*, an oncogenic translocation hotspot in lymphoid cancers, mimicking gene editing in allogeneic T cells (16). The sgRNA-expressing T cells were enriched by a CD90.1 marker (hereinafter referred to as ‘activated T cells’) and then transferred into four *H. hepaticus-*colonized 12-week-old male *Rag1^–/–^* mice with no endogenous B or T lymphocytes (Figure 1A and Supplementary Table S1).

Upon *H. hepaticus* antigen stimulation, the infused HH7-2tg T cells proliferated and differentiated into pathogenic T_H_17/T_H_1 cells, which induced inflammation in the large intestine (Figure 1A and Supplementary Figure S1A–D). Three weeks after infusion, >90% of the transferred T cells in the large intestine were sgRNA positive (Supplementary Figure S1E). The CD90.1-positive T cells selected from the gut (hereinafter referred to as ‘inflammatory T cells’) were subjected to primer extension-mediated sequencing (PEM-seq) analysis to assess editing outcomes (Figure 1A). PEM-seq is a high-throughput sequencing method that comprehensively captures genome-wide prey sequences fused to the target site, which can be used to identify uncut or perfect re-joinings, small indels, and junctions with chromosomal translocations or exogenous DNA (14,28). Of note, DNA products obtained after one-round primer extension are ligated to adapters with random molecular barcodes (RMBs); thus, PEM-seq distinguishes biological expansions from PCR duplicates, enabling comprehensively quantification of genome editing products (14,28). Over 95% of the *c-Myc* target sites were edited with indels or chromosomal translocations in both *ex vivo* activated T cells from the donor mouse and inflammatory T cells from the gut of *Rag1^–/–^* recipient mice (Figure 1B). Moreover, every type of main editing outcome remained at a similar abundance before and after infusion (Figure 1C). Correspondingly, the top five products were indels around the target site and four of them were coincident in activated and inflammatory T cells (Figure 1D). Additionally, over 93% of editing events had no impact on the coding region of the *c-Myc* gene (Supplementary Figure S2A and B). Collectively, these results indicate that the levels of various editing products persist before and after a 3-week infusion in mice.

### Chromosomal translocations in T cells undergo clonal expansion

**Figure 2.**
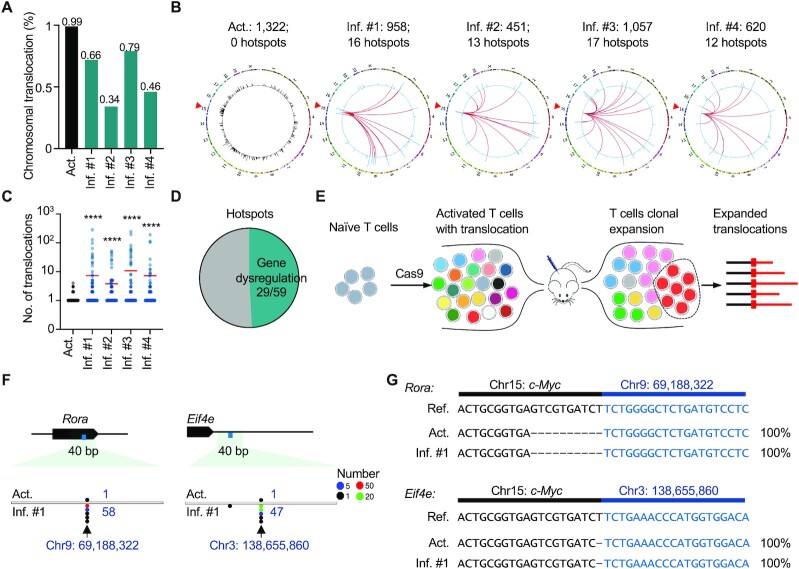
Chromosomal translocations in genome-edited T cells show TCR-independent clonal expansion *in vivo*. (**A**) Percentage of translocations among the editing events. (**B**) Circos plots showing the distribution of translocations in both activated and inflammatory T cells. The red triangles on chr15 mark the gRNA targeting site. The outer circle indicates the reference chromosomes, labeled by number or character; the inner circle presents translocations (normalized to editing events) in *c-Myc* and genome-wide in the activated (black) or inflammatory (blue) T cells. Each bar represents the number of translocations in a 1-Mb bin with a log scale. The red lines in the inflammatory T cells indicate hotspots of translocations between *c-Myc* and the indicated loci (see Materials and Methods for more details). The normalized numbers of translocations and hotspots are presented on the top. (**C**) Dot plot shows distribution of the number of junctions at each translocation site with a 1-bp bin. Each dot represents a unique translocation in one sample. Red lines show the mean value. ****P< 0.0001; two-tailed Mann–Whitney *U* test. (**D**) Proportions of translocation hotspots located in the gene regions. (**E**) Schematic showing the establishment of translocation hotspots via clonal expansion of infused T cells. (**F**) Dot plots showing the distribution of translocation junctions at the indicated sites in both activated and inflammatory T cells. 1-bp bin. (**G**) Each expanded translocation in (F) has an identical sequence in activated and inflammatory T cells.

Besides the desired editing products such as indels, chromosomal translocations were also frequently detected in Cas9-engineered T cells (4,20). By convention, chromosomal translocations are defined as junctions falling in another chromosome or outside of the ±500 kb region spanning the target site (13,28). We detected a total translocation frequency of 0.99% (1682 of 167 611 events, down to 1322 of 139 085 after normalization) in the *ex vivo* activated T cells before infusion (Figure 2A). In gut inflammatory T cells after three weeks of stimulation, the translocation levels remained at 0.34–0.79% in the four recipient mice (Figure 2A). In this context, the translocation frequency after infusion was not dramatically decreased as anticipated but was instead maintained at approximately half of that observed in the activated T cells. Translocation junctions were distributed evenly in the genome of the activated T cells, with none of the sites containing more than five junctions after normalization (Figure 2B and C, see Materials and Methods for details). In comparison, 12–17 translocation hotspots were identified in the inflammatory T cells, accompanied by decreased junctional diversity (Figure 2B and C, see Materials and Methods for details). In total, 59 translocation hotspots were identified with 5–794 junctions at the translocation site and 29 of the identified hotspots fell in the gene region, which might be capable of inducing gene dysregulation after fusion with *c-Myc* (Figure 2D). Moreover, each hotspot harbored translocation junctions at the same nucleotide in the same strand orientation (Figure 2C and Supplementary Table S2). Since translocation junctions at off-target sites routinely contained junctions in both orientations, these hotspots were possibly derived from clonal expansion of a single translocation event (Figure 2E).

To exclude the possibility that the translocation hotspots were artifacts generated by PCR duplicates, we examined the length distribution of sequencing reads extracted from the same hotspot. The *bona fide* translocation hotspots derived from clonal expansion should have diverse prey lengths generated by sonication as well as diverse RMBs introduced after one-round primer extension during library preparation (Supplementary Figure S3A). We found that these sequencing reads did show highly diverse lengths and RMBs at each site, exemplified by the junctions on chr6: 21 910 984 in the inflammatory T cells from mouse #1 (Supplementary Figure S3B and C), ruling out the possibility of PCR duplicates. We also performed PCR and Sanger sequencing analysis of three or four hotspots from each recipient mouse and found that all the tested translocation hotspots could be readily detected by PCR in the inflammatory T cells but not in the activated T cells or control naïve T cells (Supplementary Figure S3D, E, and Supplementary Table S3). Moreover, we found that two hotspots from mouse #1 had the same junction site as those observed in the activated T cells, and some hotspots occurred in at least two mice, indicating that the translocation hotspots might originate from preexisting chromosomal translocations in the activated T cells before infusion (Figure 2F, G, and Supplementary Table S2). Collectively, we concluded that the identified translocation hotspots originated from robust clonal expansion of these translocation-harboring T cells in response to *H. hepaticus* antigen stimulation.

### Viral DNA integration shows a clonal expansion pattern

**Figure 3.**
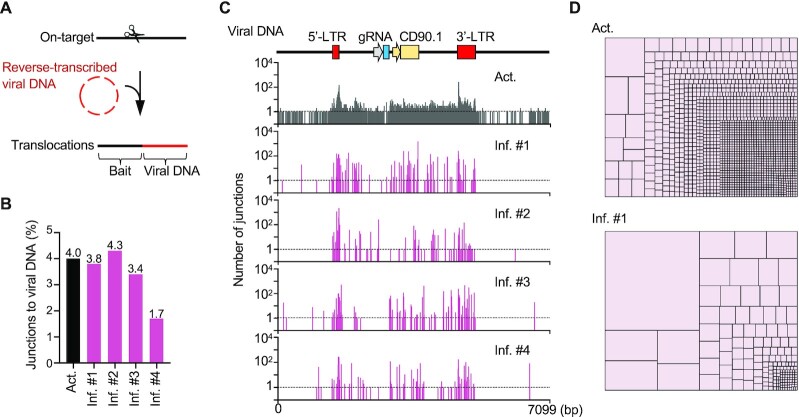
Viral DNA insertions in the edited T cells undergo clonal expansion after infusion. (**A**) Schematic of viral DNA insertions identified by PEM-seq. The prey sequences aligned to the viral genome indicate viral DNA integration. (**B**) Percentages of junctions to viral DNA among the editing events. The total editing efficiency is indicated in Figure 1B. (**C**) Distribution of junctions in the viral genome with a 1-bp bin. The functional elements of the viral DNA are shown on the top. (**D**) Tree map showing the proportion of each junction to the viral genome at a single-nucleotide resolution. Each rectangle represents a unique junction, and the area of the rectangle represents percentage of the indicated junction.

We used retrovirus, an RNA virus, to deliver the gRNA in our model (Figure 1A). Once entering T cells, the RNA spanning from 5’- to 3’-long terminal repeat (LTR) will be reverse transcribed into viral DNA (30) and integrate into CRISPR/Cas9-targeted site to generate chimeric sequences containing both targeted locus and viral DNA (Figure 3A). We analyzed the PEM-seq libraries by aligning the translocation sequences against the viral vector (13) and found that virus integrations accounted for 4% of total editing events in the activated T cells, and the frequencies remained at comparable levels for three weeks after infusion *in vivo* (Figure 3B). The virus integration sites in the activated T cells were distributed across the viral genome with 1836 junction sites, with modest enrichments in the two LTR regions (Figure 3C and Supplementary Figure S4A). The virus integration sites were also enriched in the LTRs in the inflammatory T cells, but the diversity of identified junctions in the retrovirus genome decreased greatly, and the accumulated numbers at each junction site were higher than those in the activated T cells (Figure 3C, D, Supplementary Figure S4A and B). Specifically, dozens of sites in the region between LTRs harbored hundreds of junctions after infusion for three weeks (Figure 3C). In addition, more than 50% of virus integration sites were unique in each recipient and <7% (10 out of 146) of virus integration sites were coincident in the four mice (Supplementary Figure S4C), implying a random selection of virus integrations. Collectively, these data indicate that the cells containing integrated viral DNA also experienced clonal expansion after infusion for three weeks *in vivo*.

### Large deletions and insertions expand stochastically *in vivo*

**Figure 4.**
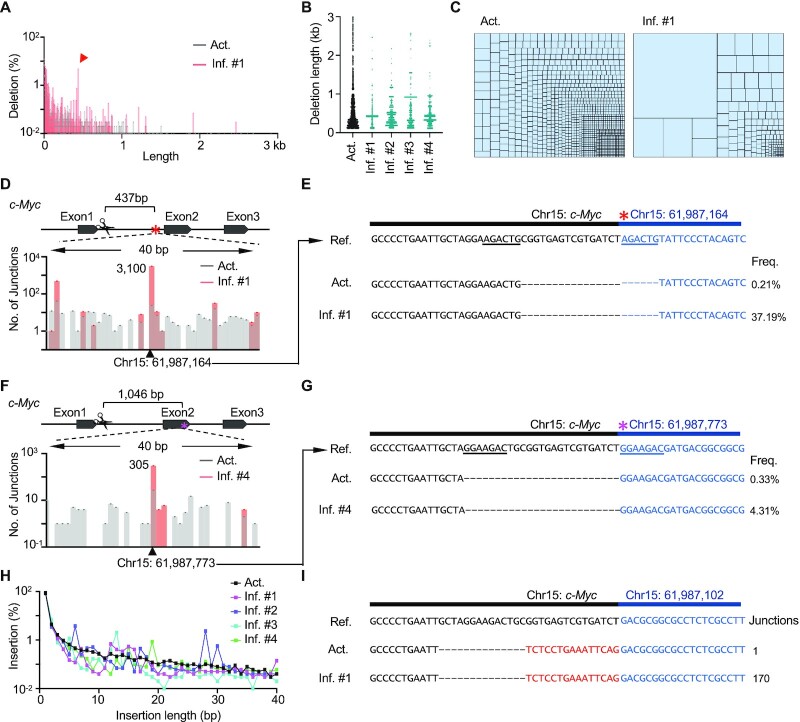
Large deletions and insertions amplify to hundreds of clones*in vivo*. (**A**) The distribution of deletions in activated and inflammatory T cells at a single-nucleotide resolution. (**B**) Distribution of large deletions with a deletion length of 100–3000 bp per 1-bp bin. Each dot represents a junction. (**C**) Tree map showing the proportion of junctions with a deletion length within 100–3000 bp. Each rectangle represents a unique large deletion with a 1-bp bin, and the area of the rectangle represents percentage of the indicated junction. (**D**) Bar plot showing the number of junctions at the indicated locus within the first intron of *c-Myc*; 1-bp bin. The black triangle and red asterisk indicate the junction of expanded large deletions. (**E**) Sequences of the expanded large deletion in (D). The reference (ref.) sequence contains the bait sequence (black) and expected prey junction (blue). The underlines show the micro homologous sequence. Deletions (‘–’) are marked. Frequencies (freq.) show the relative percentages of the indicated products among the total number of large deletions. (**F**) Bar plot showing the number of junctions at the indicated locus in the second exon of *c-Myc* in the activated and inflammatory T cells from mouse #4. The black triangle and magenta asterisk indicate the junction of expanded large deletions. (**G**) Sequences of the expanded large deletion in (F). Legends are depicted as described in (E). (**H**) Distribution of insertions with different lengths in activated and inflammatory T cells. (**I**) An example of an expanded insertion that has 1 or 170 hits in activated or inflammatory T cells from mouse #2, respectively.

The vast majority of target mutations induced by CRISPR/Cas9 were deletions and insertions (Figure 1C). Regarding deletions, the relative percentages of small (≤100 bp) and large (101 bp to 500 kb) deletions remained constant, respectively, before and after infusion for three weeks (Supplementary Figure S5A). Most of the deletions fell in the 1-kb regions downstream of the target site, with a declining trend over the length of that region in activated T cells (Figure 4A), while fluctuations in both proximal and distal regions were observed in the inflammatory T cells (Figure 4A, B, and Supplementary Figure S5B). Correspondingly, the infused T cells exhibited lower junction diversity and harbored more deletions per junction site than the activated T cells; the average number of deletions at each site was elevated up to 5-fold after infusion (Figure 4C and Supplementary Figure S5C–E). For instance, two highly expanded large deletions accounted for 37.19% and 4.31% of large deletions in inflammatory T cells from recipient #1 and #4, respectively (Figure 4D–G). Of note, different highly-expanded large deletions were distinct in four recipients (Figure 4B and Supplementary Figure S5B), indicating a stochastic expansion of large deletions induced by Cas9 cleavage. Besides large deletions, small deletions clonally expanded after infusion as well, exemplified by a 61-bp deletion in mouse #2 (Supplementary Figure S5B, F and G).

Insertions accounted for over 20% of the editing events (Figure 1C). Similar to those of deletions, the frequencies of insertions also exhibited a declining trend over length in both activated and inflammatory T cells (Figure 4H). Among all the insertions, a 1-bp T insertion accounted for >73% in both activated and inflammatory T cells (Supplementary Figure S5H). Due to clonal expansion, pileups of insertion junctions with irregular length were observed in inflammatory T cells, while the length distribution curve of insertions was relatively smooth in the activated T cells (Figure 4H and Supplementary Figure S5I). Specifically, a 15-bp insertion at the junction of chr15:61 987 102 was enriched by up to 170 copies in the inflammatory T cells from mouse #2 (Figure 4I and Supplementary Figure S5I).

### Cas9-induced SVs persist for two months

**Figure 5.**
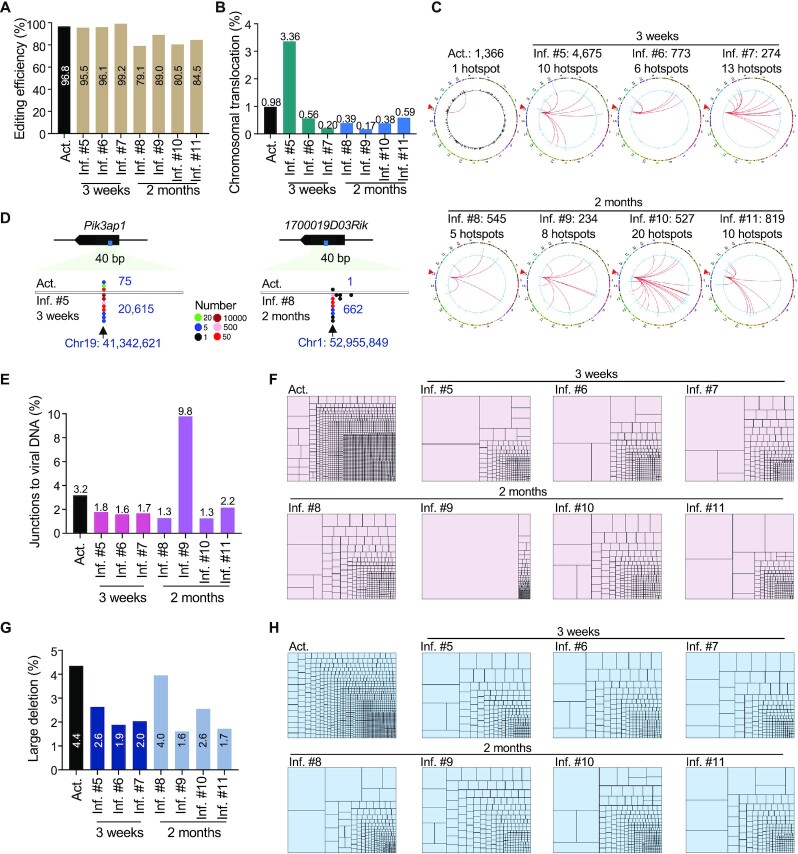
Persistence and stochastic expansion of SVs in the second batch of infused T cells for 2 months. (**A**) Editing efficiency of CRISPR/Cas9 targeting *c-Myc* in activated and inflammatory T cells. (**B**) Percentage of genomic translocations among the editing events. (**C**) Circos plots showing the distribution of translocations in activated and inflammatory T cells. Legends are described in Figure 2B. (**D**) Dot plots showing the distribution of translocation junctions at the indicated sites in activated and inflammatory T cells. 1-bp bin. Legends of dot plots are indicated in the middle. (**E**) Percentage of junctions to viral DNA among the editing events. (**F**) Tree map showing the proportion of each junction to the viral genome at a single-nucleotide resolution. Each rectangle represents a unique junction site, and the area of the rectangle represents its percentage. (**G**) Percentage of large deletions among the editing events. (**H**) Tree map showing the proportion of each junction to the large deletions at a single-nucleotide resolution. Each rectangle represents a unique junction site, and the area of the rectangle represents its percentage.

To investigate the long-term progression of Cas9-induced SVs *in vivo*, we generated another batch of experiment and kept the inflammatory recipients for as long as 2 months after T cell infusion (Supplementary Figure S6A and Figure 1A). Consistently, CRISPR/Cas9 efficiently targeted the *c-Myc* locus and generated chromosomal translocations at a frequency of 0.98% in *ex-vivo* activated T cells (Figure 5A and B). Chromosomal translocations presented in the inflammatory T cells 3 weeks post infusion, and notably persisted for even 2 months at levels from 0.17 to 0.59% (Figure 5B). One junction hotspot was detected in activated T cells, while 5–20 hotspots were observed in the recipients, indicating a clonal expansion as previously observed (Figure 5C and Supplementary Table S4). Of note, we found that the inflammatory T cells from a 3-week-infusion recipient (Inf. #5) had a level of translocation at ∼3.36%, much higher than 0.98% in *ex-vivo* activated T cells. Remarkably, 72.2% of identified translocation junctions in Inf. #5 fell in a hotspot on the *Pik3ap1* gene, which was extensively expanded from the only hotspot in activated T cells (Figure 5B–D). Ten translocation hotspots from 2-month-infusion mice also contained junctions in activated T cells due to clonal expansion, exemplified by the hotspot on *1700019D03Rik* from Inf. #8 (Figure 5D and Supplementary Table S4). No translocation hotspot was coincident in the two batches; while one translocation hotspot on *Agps* was observed in both Inf. #7 (3-week infusion) and Inf. #8 (2-month infusion) in the second batch (Supplementary Tables S2 and S4), suggesting a stochastic selection of chromosomal translocations *in vivo*.

Similar to chromosomal translocations, viral DNA integrations in 2-month-infusion mice also showed clonal expansion patterns similar to those with 3-week-infusion (Figure 5E, F, and Supplementary Figure S6B). Specifically, an integration at 3’-LTR expanded to 30 401 (down to 12 112 after normalization) clones after 2-month infusion and accounted for 89.1% of identified viral DNA integrations in Inf. #8, causing a 2-fold increase of total viral insertions (Figure 5E and Supplementary Figure S6B). Moreover, the percentages of large deletions in inflammatory T cells after 2-month infusion were comparable to those for 3-week infusion and both were lower than that of activated T cells (Figure 5G, H, and Supplementary Figure S6C). Collectively, these data indicate that SVs persist and undergo substantial and stochastic clonal expansion in the infused T cells for at least 2 months *in vivo*.

## DISCUSSION

In this study, we used a chronic inflammation mouse model with unique TCR-Tg T cells that specifically recognize an *H. hepaticus* antigen (29). The colonization of *H. hepaticus* stimulates TCR-Tg T cells, similar to other TCR T and CAR T cells that are activated by tumor cells. We employed the high-throughput sequencing method PEM-seq to trace TCR-Tg T cells from activation to inflammation and presented a comprehensive profile of the progression of SVs induced by gene editing. Unexpectedly, the SVs did not vanish by themselves during proliferation selection under inflammation but persisted at a relatively high level *in vivo* for three weeks and two months post-editing (Figures 2A, 3B, 5B, 5E, and G). Recently, reports showed that infused T cells persist for up to 9 months or even decades in CAR T clinical trials (4,7). Dozens of SV-harboring T cells could expand hundreds of fold under antigen stimulation, and SVs did not appear to be involved in the expansion process (Figures 2B, C, and 5C). In this context, the proliferation of T cells could be stimulated by cytokines, independent of the TCR, and the mechanism of selection of TCR or CAR T cells upon antigen stimulation remains a puzzle (9,31). Therefore, SVs are likely passengers but not the driving force for the selection of TCR T cells in our tested cases. However, it is possible that some cells may gain a proliferation advantage if their CRISPR/Cas9-induced SVs possess oncogenic properties. In addition, no significant difference of translocation frequencies was detected between male and female mice after a 3-week infusion and SVs-harboring T cells in female recipients also underwent clonal expansions (Supplementary Figure S7A–D and Table S1), excluding the gender influence on SV persistence and expansion.

Our observations may shed light on the development of oncogenic chromosomal translocations in T or B lymphocytes. In this study, T cells stably expressing Cas9 were edited at *c-Myc* by retrovirus-delivered gRNA, which is not favorable for clinical settings for CAR or TCR T therapy. However, the way to deliver Cas9 and gRNA might alter the formation of SVs and barely affect *in vivo* progression of SVs after infusion. Moreover, *c-Myc*-mediated oncogenesis on the persistent and expanded SVs was not observed in this study. Therefore, our findings also raise concerns about the safety of CRISPR/Cas9-edited T cells meditated immunotherapy. Persistent SVs might be a problem for CRISPR/Cas9-edited TCR T cells or similar CAR T cells, as these SV-containing cells may gain more mutations during further clonal expansion. To circumvent potential harm of SVs, new gene editing strategies that either eliminate chromosomal abnormalities, such as Cas9TX (20), or avoid DSB generation, such as base editors or prime editors (32), should be applied to reduce SVs before large-scale transfer of gene-edited T cells into patients. Efficient delivery of editor protein and gRNA by Ribonucleoprotein (RNPs) instead of virus should also be used to avoid viral DNA integration as well (33). In addition, it is essential to trace the long-term persistence of SVs after infusion in patients, as randomly expanded SVs cannot be predicted from *ex vivo* engineered T cells.

## DATA AVAILABILITY

All sequencing data presented in the present study were deposited in the NCBI Gene Expression Omnibus (GEO) under the accession number GSE202887 (https://www.ncbi.nlm.nih.gov/geo/query/acc.cgi?acc=GSE202887). The PEM-Q pipeline is available at https://github.com/JiazhiHuLab.

## Supplementary Material

gkac887_Supplemental_File

## References

[1] Boyiadzis M.M. , Dhodapkar M.V., Brentjens R.J., Kochenderfer J.N., Neelapu S.S., Maus M.V., Porter D.L., Maloney D.G., Grupp S.A., Mackall C.L. et al. Chimeric antigen receptor (CAR) t therapies for the treatment of hematologic malignancies: clinical perspective and significance. J. Immunother. Cancer. 2018; 6:137. 30514386 10.1186/s40425-018-0460-5 PMC6278156

[2] Laskowski T. , Rezvani K. Adoptive cell therapy: living drugs against cancer. J. Exp. Med. 2020; 217:e20200377. 33227136 10.1084/jem.20200377 PMC7686916

[3] Mullard A. FDA approves fourth CAR-T cell therapy. Nat. Rev. Drug Discov. 2021; 20:166. 10.1038/d41573-021-00031-9 33574586

[4] Stadtmauer E.A. , Fraietta J.A., Davis M.M., Cohen A.D., Weber K.L., Lancaster E., Mangan P.A., Kulikovskaya I., Gupta M., Chen F. et al. CRISPR-engineered t cells in patients with refractory cancer. Science. 2020; 367:eaba7365. 32029687 10.1126/science.aba7365 PMC11249135

[5] Styczyński J. A brief history of CAR-T cells: from laboratory to the bedside. Acta Haematol. Pol. 2020; 51:2–5.

[6] Stock S. , Schmitt M., Sellner L. Optimizing manufacturing protocols of chimeric antigen receptor t cells for improved anticancer immunotherapy. Int. J. Mol. Sci. 2019; 20:6223. 10.3390/ijms20246223 PMC6940894 31835562

[7] Melenhorst J.J. , Chen G.M., Wang M., Porter D.L., Chen C., Collins M.A., Gao P., Bandyopadhyay S., Sun H., Zhao Z. et al. Decade-long leukaemia remissions with persistence of CD4(+) CAR t cells. Nature. 2022; 602:503–509. 35110735 10.1038/s41586-021-04390-6 PMC9166916

[8] Biasco L. , Izotova N., Rivat C., Ghorashian S., Richardson R., Guvenel A., Hough R., Wynn R., Popova B., Lopes A. et al. Clonal expansion of t memory stem cells determines early anti-leukemic responses and long-term CAR t cell persistence in patients. Nat Cancer. 2021; 2:629–642. 34345830 10.1038/s43018-021-00207-7 PMC7611448

[9] Sheih A. , Voillet V., Hanafi L.A., DeBerg H.A., Yajima M., Hawkins R., Gersuk V., Riddell S.R., Maloney D.G., Wohlfahrt M.E. et al. Clonal kinetics and single-cell transcriptional profiling of CAR-T cells in patients undergoing CD19 CAR-T immunotherapy. Nat. Commun. 2020; 11:219. 31924795 10.1038/s41467-019-13880-1 PMC6954177

[10] Liu X. , Zhang Y., Cheng C., Cheng A.W., Zhang X., Li N., Xia C., Wei X., Liu X., Wang H. CRISPR-Cas9-mediated multiplex gene editing in CAR-T cells. Cell Res. 2017; 27:154–157. 27910851 10.1038/cr.2016.142 PMC5223227

[11] Ren J. , Liu X., Fang C., Jiang S., June C.H., Zhao Y. Multiplex genome editing to generate universal CAR t cells resistant to PD1 inhibition. Clin. Cancer Res. 2017; 23:2255–2266. 27815355 10.1158/1078-0432.CCR-16-1300 PMC5413401

[12] Eyquem J. , Mansilla-Soto J., Giavridis T., van der Stegen S.J., Hamieh M., Cunanan K.M., Odak A., Gonen M., Sadelain M. Targeting a CAR to the TRAC locus with CRISPR/Cas9 enhances tumour rejection. Nature. 2017; 543:113–117. 28225754 10.1038/nature21405 PMC5558614

[13] Liu M. , Zhang W., Xin C., Yin J., Shang Y., Ai C., Li J., Meng F.-L., Hu J. Global detection of DNA repair outcomes induced by CRISPR–Cas9. Nucleic Acids Res. 2021; 49:8732–8742. 34365511 10.1093/nar/gkab686 PMC8421148

[14] Yin J. , Liu M., Liu Y., Wu J., Gan T., Zhang W., Li Y., Zhou Y., Hu J. Optimizing genome editing strategy by primer-extension-mediated sequencing. Cell Discov. 2019; 5:18. 30937179 10.1038/s41421-019-0088-8 PMC6434046

[15] Saha K. , Sontheimer E.J., Brooks P.J., Dwinell M.R., Gersbach C.A., Liu D.R., Murray S.A., Tsai S.Q., Wilson R.C., Anderson D.G. et al. The NIH somatic cell genome editing program. Nature. 2021; 592:195–204. 33828315 10.1038/s41586-021-03191-1 PMC8026397

[16] Kuppers R. , Dalla-Favera R. Mechanisms of chromosomal translocations in b cell lymphomas. Oncogene. 2001; 20:5580–5594. 11607811 10.1038/sj.onc.1204640

[17] Nussenzweig A. , Nussenzweig M.C. Origin of chromosomal translocations in lymphoid cancer. Cell. 2010; 141:27–38. 20371343 10.1016/j.cell.2010.03.016 PMC2874895

[18] Frock R.L. , Hu J., Meyers R.M., Ho Y.J., Kii E., Alt F.W. Genome-wide detection of DNA double-stranded breaks induced by engineered nucleases. Nat. Biotechnol. 2015; 33:179–186. 25503383 10.1038/nbt.3101 PMC4320661

[19] Hu J. , Meyers R.M., Dong J., Panchakshari R.A., Alt F.W., Frock R.L. Detecting DNA double-stranded breaks in mammalian genomes by linear amplification-mediated high-throughput genome-wide translocation sequencing. Nat. Protoc. 2016; 11:853–871. 27031497 10.1038/nprot.2016.043 PMC4895203

[20] Yin J. , Lu R., Xin C., Wang Y., Ling X., Li D., Zhang W., Liu M., Xie W., Kong L. et al. Cas9 exo-endonuclease eliminates chromosomal translocations during genome editing. Nat. Commun. 2022; 13:1204. 35260581 10.1038/s41467-022-28900-w PMC8904484

[21] Sheridan C. Off-the-shelf, gene-edited CAR-T cells forge ahead, despite safety scare. Nat. Biotechnol. 2022; 40:5–8. 34912036 10.1038/d41587-021-00027-1

[22] Jo S. , Das S., Williams A., Chretien A.S., Pagliardini T., Le Roy A., Fernandez J.P., Le Clerre D., Jahangiri B., Chion-Sotinel I. et al. Endowing universal CAR T-cell with immune-evasive properties using TALEN-gene editing. Nat. Commun. 2022; 13:3453. 35773273 10.1038/s41467-022-30896-2 PMC9247096

[23] Yin J. , Hu J. The origin of unwanted editing byproducts in gene editing. Acta Biochim. Biophy. Sin. 2022; 54:767–781. 10.3724/abbs.2022056 PMC9827802 35643959

[24] Kosicki M. , Tomberg K., Bradley A. Repair of double-strand breaks induced by CRISPR-Cas9 leads to large deletions and complex rearrangements. Nat. Biotechnol. 2018; 36:765–771. 30010673 10.1038/nbt.4192 PMC6390938

[25] Hanlon K.S. , Kleinstiver B.P., Garcia S.P., Zaborowski M.P., Volak A., Spirig S.E., Muller A., Sousa A.A., Tsai S.Q., Bengtsson N.E. et al. High levels of AAV vector integration into CRISPR-induced DNA breaks. Nat. Commun. 2019; 10:4439. 31570731 10.1038/s41467-019-12449-2 PMC6769011

[26] Shah N.N. , Qin H., Yates B., Su L., Shalabi H., Raffeld M., Ahlman M.A., Stetler-Stevenson M., Yuan C., Guo S. et al. Clonal expansion of CAR t cells harboring lentivector integration in the CBL gene following anti-CD22 CAR T-cell therapy. Blood Adv. 2019; 3:2317–2322. 31387880 10.1182/bloodadvances.2019000219 PMC6693002

[27] Hoijer I. , Emmanouilidou A., Ostlund R., van Schendel R., Bozorgpana S., Tijsterman M., Feuk L., Gyllensten U., den Hoed M., Ameur A CRISPR-Cas9 induces large structural variants at on-target and off-target sites in vivo that segregate across generations. Nat. Commun. 2022; 13:627. 35110541 10.1038/s41467-022-28244-5 PMC8810904

[28] Liu Y. , Yin J., Gan T., Liu M., Xin C., Zhang W., Hu J. PEM-seq comprehensively quantifies DNA repair outcomes during gene-editing and DSB repair. STAR Protoc. 2022; 3:101088. 35462794 10.1016/j.xpro.2021.101088 PMC9019705

[29] Xu M. , Pokrovskii M., Ding Y., Yi R., Au C., Harrison O.J., Galan C., Belkaid Y., Bonneau R., Littman D.R. c-MAF-dependent regulatory T cells mediate immunological tolerance to a gut pathobiont. Nature. 2018; 554:373–377. 29414937 10.1038/nature25500 PMC5814346

[30] Stoye J.P. Studies of endogenous retroviruses reveal a continuing evolutionary saga. Nat. Rev. Microbiol. 2012; 10:395–406. 22565131 10.1038/nrmicro2783

[31] Vella A.T. , Dow S., Potter T.A., Kappler J., Marrack P. Cytokine-induced survival of activated T cells in vitro and in vivo. Proc. Natl. Acad. Sci. U.S.A. 1998; 95:3810–3815. 9520449 10.1073/pnas.95.7.3810 PMC19919

[32] Anzalone A.V. , Koblan L.W., Liu D.R. Genome editing with CRISPR–Cas nucleases, base editors, transposases and prime editors. Nat. Biotechnol. 2020; 38:824–844. 32572269 10.1038/s41587-020-0561-9

[33] Wang H.X. , Li M., Lee C.M., Chakraborty S., Kim H.W., Bao G., Leong K.W. CRISPR/Cas9-based genome editing for disease modeling and therapy: challenges and opportunities for nonviral delivery. Chem. Rev. 2017; 117:9874–9906. 28640612 10.1021/acs.chemrev.6b00799

