# Supplementary material for: CRISPR/Cas9-induced structural variations expand in T lymphocytes in vivo
Source: Nucleic Acids Res. 2022 Oct 16;50(19):11128–37. doi: 10.1093/nar/gkac887 (PMC9638931; doi:10.1093/nar/gkac887)
Supplement: gkac887_Supplemental_File [file gkac887_supplemental_file.pdf]

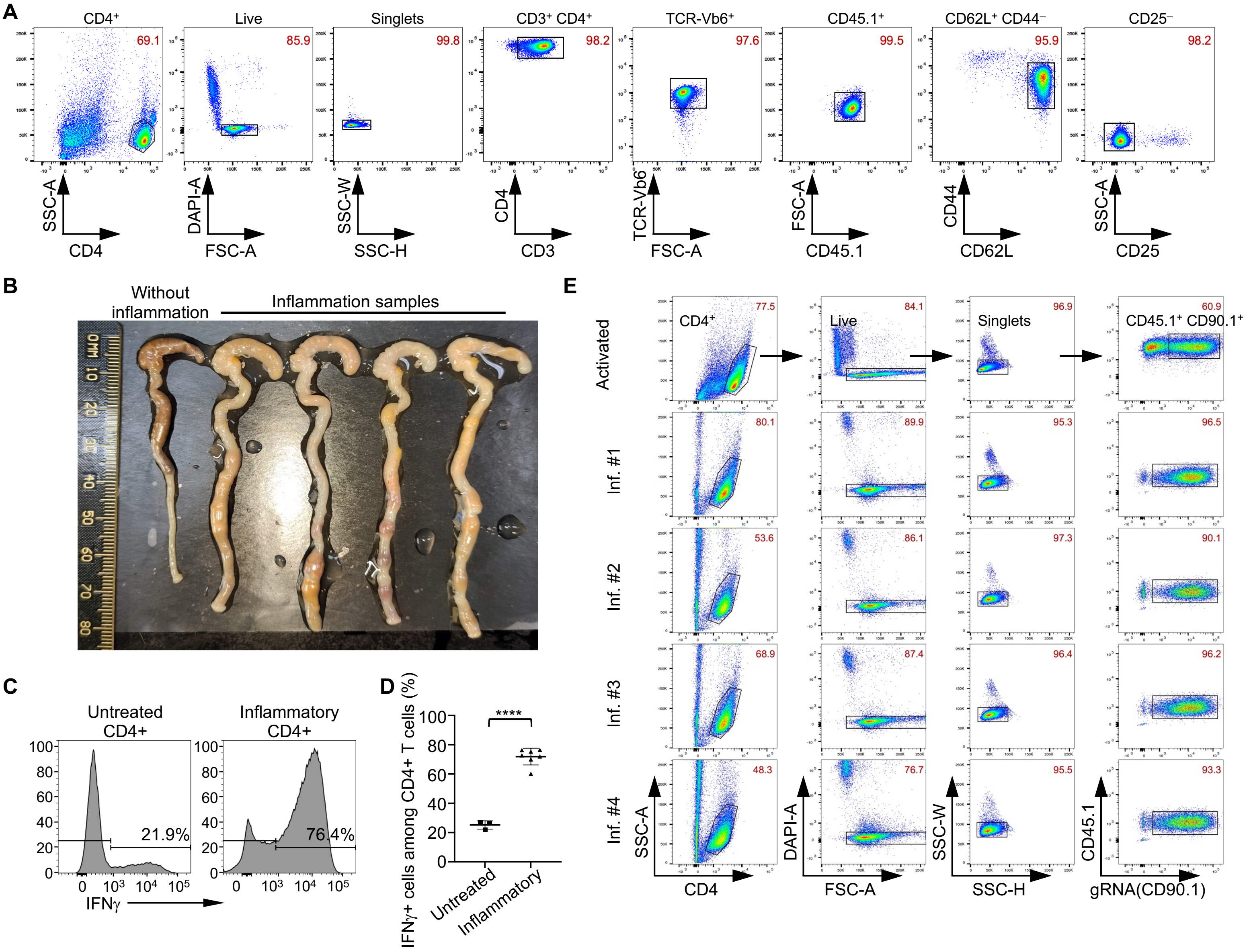

Fig. S1 Cell sorting strategies used in this study.

A. The gating strategies for isolating naïve T cells.

B. Intestines of *Rag1*$^{-/-}$ mice with or without *Helicobacter hepaticus*-induced inflammation.

C. FACS analysis of the expression level of IFNγ in the CD4+ T cells isolated from control and inflammatory mice.

D. Percentages of IFNγ expressing cells among the CD4+ T cells isolated from control and inflammatory mice. ****, $p<0.0001$; two-tailed *t*-test.

E. Gating strategies for sorting CRISPR/Cas9-edited T cells before or after infusion.

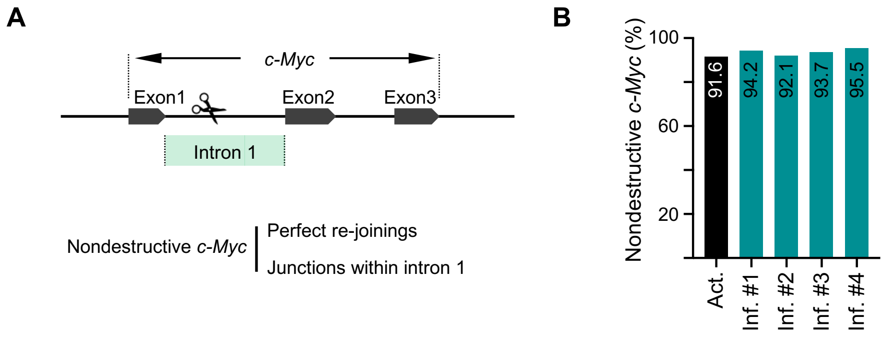

Fig. S2 The impact of gene editing on *c-Myc*.

A. Schematic showing the editing events that would not cause *c-Myc* disruption.

B. Percentage of events that would not disrupt *c-Myc*.

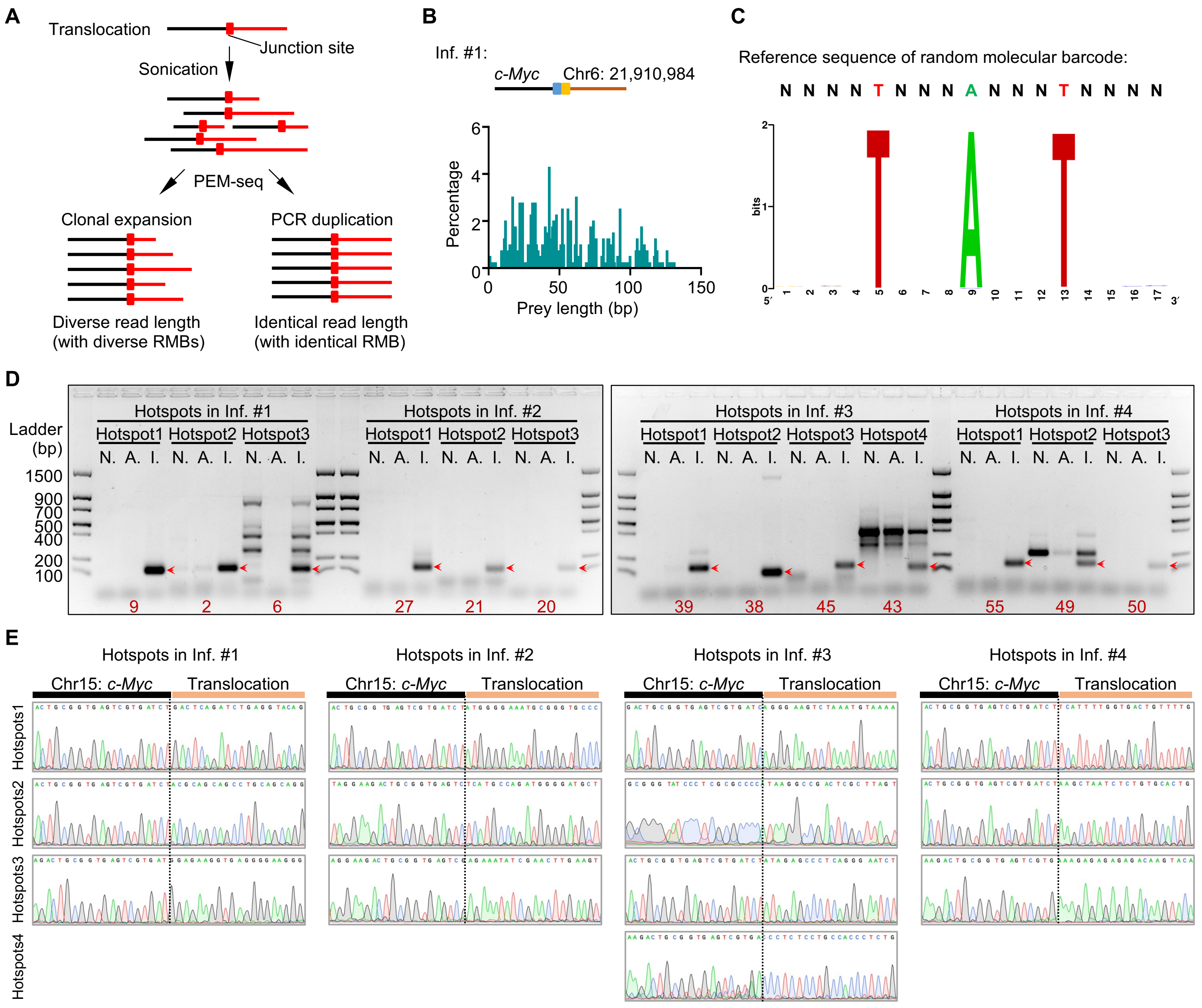

Fig. S3 Chromosomal translocations in genome-edited T cells show clonal expansion *in vivo*.

A. Schematic of translocation expansion identified by PEM-seq. After sonication and PEM-seq, the *bona fide* expanded junction has an identical bait sequence, the same junction site, diverse prey lengths, and different random molecular barcodes (RMBs). However, translocations from PCR duplication have the same read length, sequence, and RMB.

B. The prey length distribution of an identified translocation hotspot in inflammatory #1 T cells. Top: Schematic of one expanded translocation. Details are described in Methods.

C. Sequence logos showing the conserved sequence of RMBs from reads in (**B**). Top: The reference RMB used in this study.

D. Agarose gel electrophoresis showing PCR products detected by primer sets (listed in Table S3) targeting the indicated translocations in each sample. N., naïve T cells; A., activated T cells; I., inflammatory T cells. The red arrows show the expected products for desired translocations. The red numbers on the bottom show the “Serial No.” of hotspots listed in Table S2.

E. Sanger sequencing to validate the indicated translocations shown in (**D**).

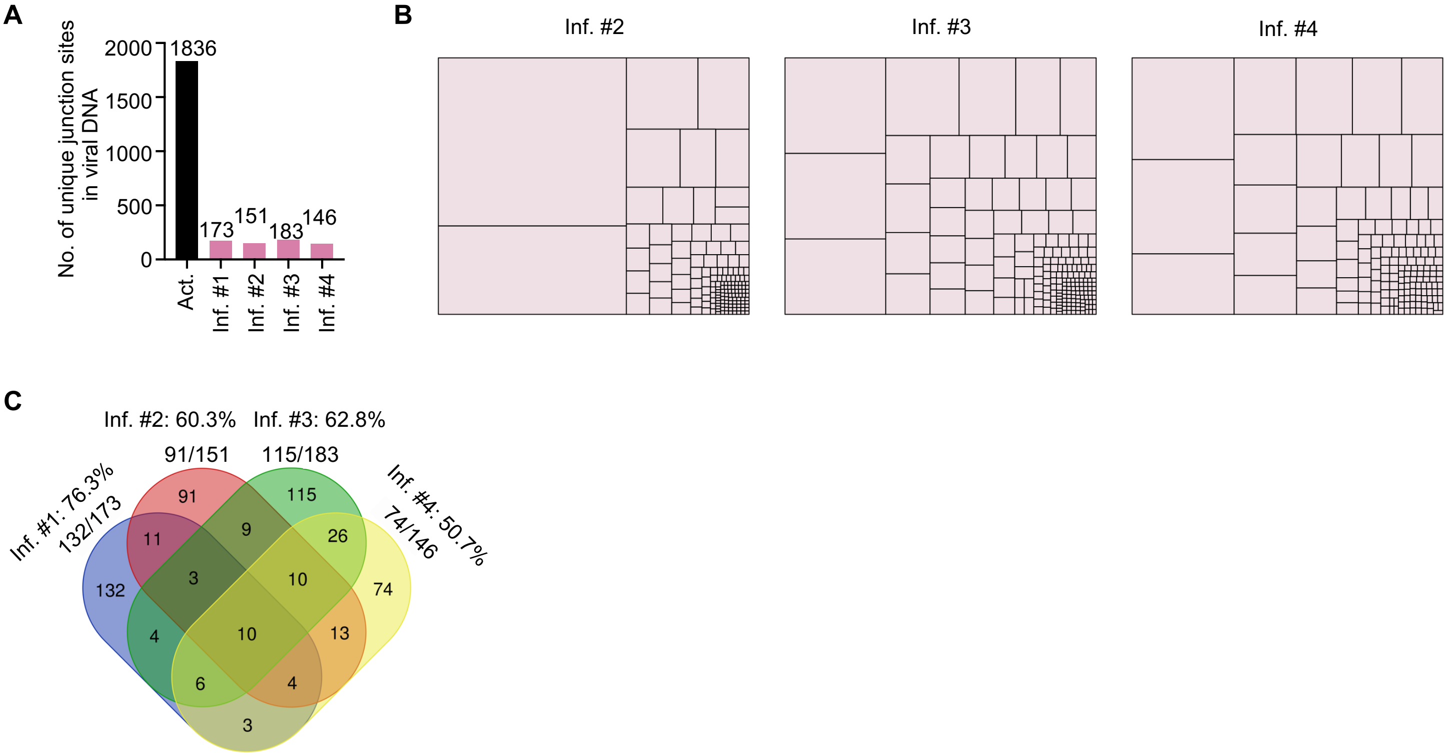

Fig. S4 Clonal expansion of T cells harboring viral DNA integration.

A. Number of unique junction sites in viral DNA.

B. Tree map showing the proportion of each junction to the viral genome at a single-nucleotide resolution. Legends are depicted as described in Fig. 3**D**.

C. Venn plot showing the overlapped viral DNA integration in the four recipients. Percentages indicate the proportions of unique viral DNA integration in each mouse.

A

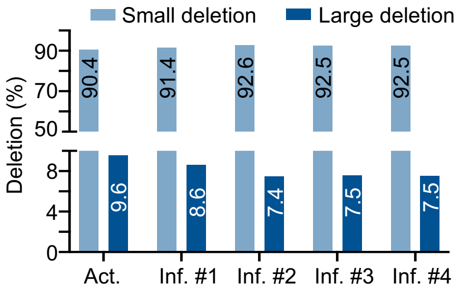

B

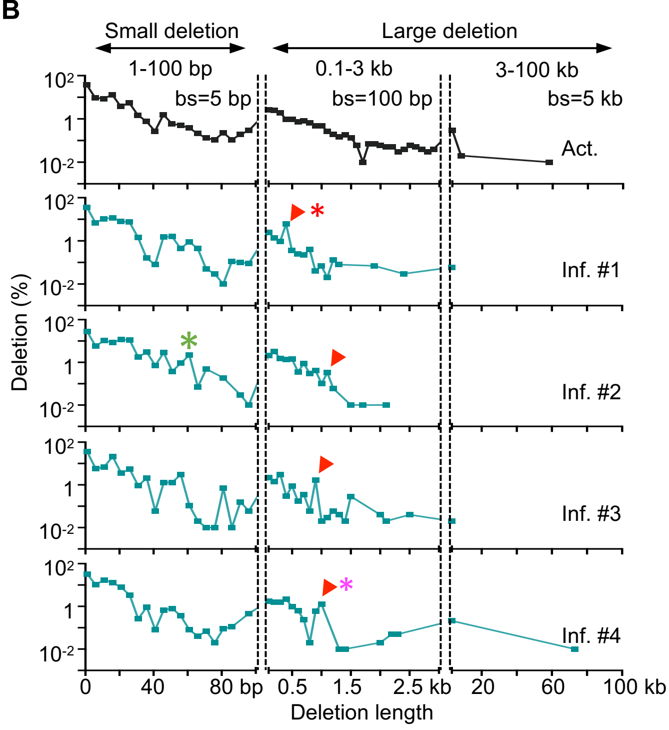

C

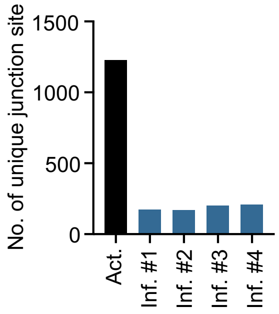

D

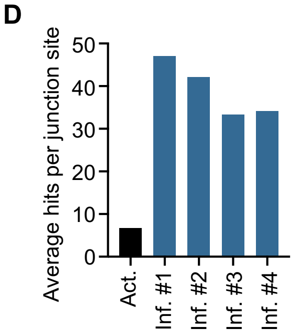

E

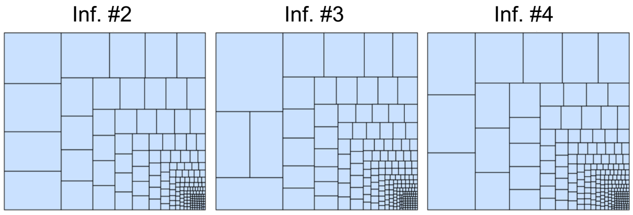

F

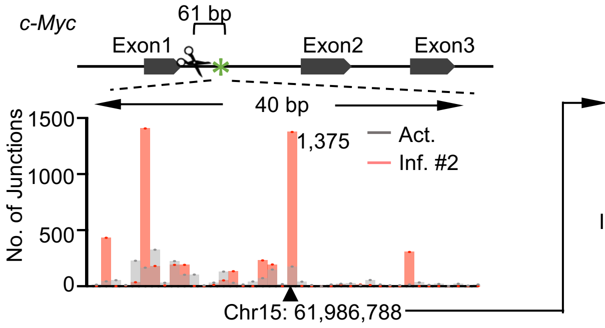

G

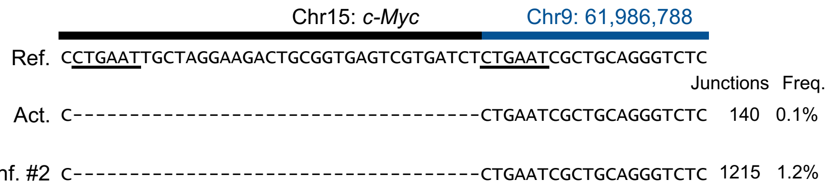

H

Table 1. Frequency of 1-bp insertion before or after infusion.

Insertion sequence	Act.	Inf. #1	Inf. #2	Inf. #3	Inf. #4
T	74.9%	77.2%	73.8%	80.6%	78.2%
G	7.3%	9.8%	3.6%	3.6%	4.3%
A	2.8%	0.9%	1.4%	0.9%	2.7%
C	1.8%	1.1%	4.2%	1.1%	1.5%

I

Table 2. Frequency of insertions (> 1 bp) in the edited T cells.

Insertion sequence	Insertion length	Act.	Inf. 1#	Inf. 2#	Inf. 3#	Inf. 4#
TG	2	0.812%	0.858%	0.357%	1.508%	0.084%
TT	2	0.661%	0.216%	0.268%	0.190%	0.883%
TA	2	0.578%	0.018%	0.244%	0.752%	0.094%
GG	2	0.390%	0.096%	0.143%	0.063%	0.080%
TC	2	0.359%	1.322%	0.320%	0.462%	0.034%
AA	2	0.281%	0.021%	0.017%	0.027%	0.121%
CA	2	0.203%	0.825%	0.366%	0.028%	2.560%
GA	2	0.198%	0.386%	0.055%	0.159%	0.090%
CC	2	0.161%	0.038%	0.081%	0.302%	0.157%
CG	2	0.161%	0.075%	0.061%	0.138%	0.124%
AG	2	0.125%	0.027%	0.022%	0.015%	0.044%
CT	2	0.117%	0.225%	1.055%	0.059%	0.084%
GC	2	0.099%	0.015%	0.053%	0.051%	0.037%
AC	2	0.094%	0.015%	0.029%	3.002%	0.070%
GT	2	0.086%	0.129%	0.390%	0.030%	0.877%
TGG	3	0.107%	0.007%	0.018%	0.086%	0.013%
TCT	3	0.081%	0.001%	0.309%	0.004%	0.010%
TCA	3	0.049%	0.049%	0.011%	0.000%	0.455%
CAG	3	0.036%	0.025%	0.002%	0.003%	0.084%
ACT	3	0.036%	0.231%	0.009%	0.004%	0.003%
GAG	3	0.029%	0.001%	0.018%	0.144%	0.044%
ACC	3	0.026%	0.015%	0.357%	0.003%	0.000%
CGG	3	0.018%	0.006%	0.000%	0.109%	0.003%
ACCG	4	0.013%	0.000%	0.000%	0.213%	0.044%
TCC	3	0.010%	0.189%	0.028%	0.001%	0.345%
CGTCTTCCCTCTC	13	0.008%	0.157%	0.000%	0.000%	0.000%
GGGA	4	0.008%	0.000%	0.290%	0.000%	0.000%
AGTCT	5	0.005%	0.000%	0.022%	0.000%	0.094%
AAACT	5	0.003%	0.001%	0.002%	0.992%	0.010%
ACCTAG	6	0.003%	0.000%	2.972%	0.001%	0.010%
TCTCCTGAAATTCAG	15	0.003%	0.392%	0.002%	0.000%	0.003%
ACGAAAGGGGGCG	13	0.000%	0.000%	0.000%	1.826%	0.040%
AGACCGTAACAGCTGCTAC CCTCGGCGG	28	0.000%	0.000%	1.880%	0.000%	0.007%
AGAGGGAGA	9	0.000%	0.000%	0.232%	0.000%	0.000%
AGCAGCTGTTACGTGAT	17	0.000%	0.000%	0.358%	0.000%	0.000%
AGTCGGC	7	0.000%	0.341%	0.002%	0.000%	0.000%
ATCCCA	6	0.000%	0.000%	0.267%	0.000%	0.000%
CAAAGGAAGGAAGG	14	0.000%	0.256%	0.000%	0.000%	0.000%
CAACGAATCGGTCACA	16	0.000%	0.297%	0.000%	0.000%	0.000%
CACTATTTTTTT	12	0.000%	0.582%	0.004%	0.000%	0.000%
CGCTAGGAAGACC	13	0.000%	0.182%	0.000%	0.000%	0.000%
GCCCTATCTCAAAACATCA	19	0.000%	0.000%	0.000%	0.000%	0.636%
GGCGAAGCCCTGCCCTTCA GGAGGCAGGAG	30	0.000%	0.000%	0.333%	0.000%	0.003%
GTGAGTGGTGAGTCG	15	0.000%	0.001%	0.000%	0.001%	0.351%
TAAAAGGCTCAGG	13	0.000%	0.000%	0.000%	0.095%	0.000%
TAAACCGCGA	10	0.000%	0.000%	0.000%	0.088%	0.000%
TAGTGCTAGGAA	12	0.000%	0.000%	0.000%	0.324%	0.000%
TCAT	4	0.000%	0.000%	0.000%	0.000%	0.201%
TTCCTAGCAGTCTTCCTAGC AATTTCCTAGGAAATTGCTA GGAAGACT	48	0.000%	0.000%	0.000%	0.000%	0.107%

Fig. S5 Deletions and insertions experience clonal expansion *in vivo*. (Legends on the next page)

Fig. S5 Deletions and insertions experience clonal expansion *in vivo*.

A. Percentages of small deletions (1-100 bp) and large deletions (0.1-500 kb) among all deletion events.

B. Distribution of deletions in the edited T cells. The number of total deletion junctions for each sample was normalized to editing events. Red arrows show the expanded deletion events. Red, magenta, and green asterisk indicates the 437-bp, 1046-bp, and 61-bp deletion event in Figs. 4**D**, 4**F**, and S5**F**, respectively. Bs, bin size.

C. Number of unique junction sites with a deletion length within 100-3000 bp.

D. Average hits per junction site with a deletion length within 100-3000 bp.

E. Tree map showing the proportion of junctions with a deletion length within 100-3000 bp in inflammatory mice #2, #3, and #4. Legends are depicted as described in Fig. 4**C**.

F. Bar plot showing the number of junctions at the indicated locus with a 61-bp small deletion; 1-bp bin. The black triangle and green asterisk indicate the junction of expanded small deletions.

G. Sequences of the expanded small deletion in (**F**). Legends are depicted as described in Fig. 4**E**.

H. Frequency of 1-bp insertion. The color scale shows the distribution of insertion frequency, and the red marks the top insertions in each mouse.

I. Frequency of insertions (>1 bp) in activated and inflammatory T cells. The color scale shows the distribution of insertion frequency, and orange marks the top insertion in each sample.

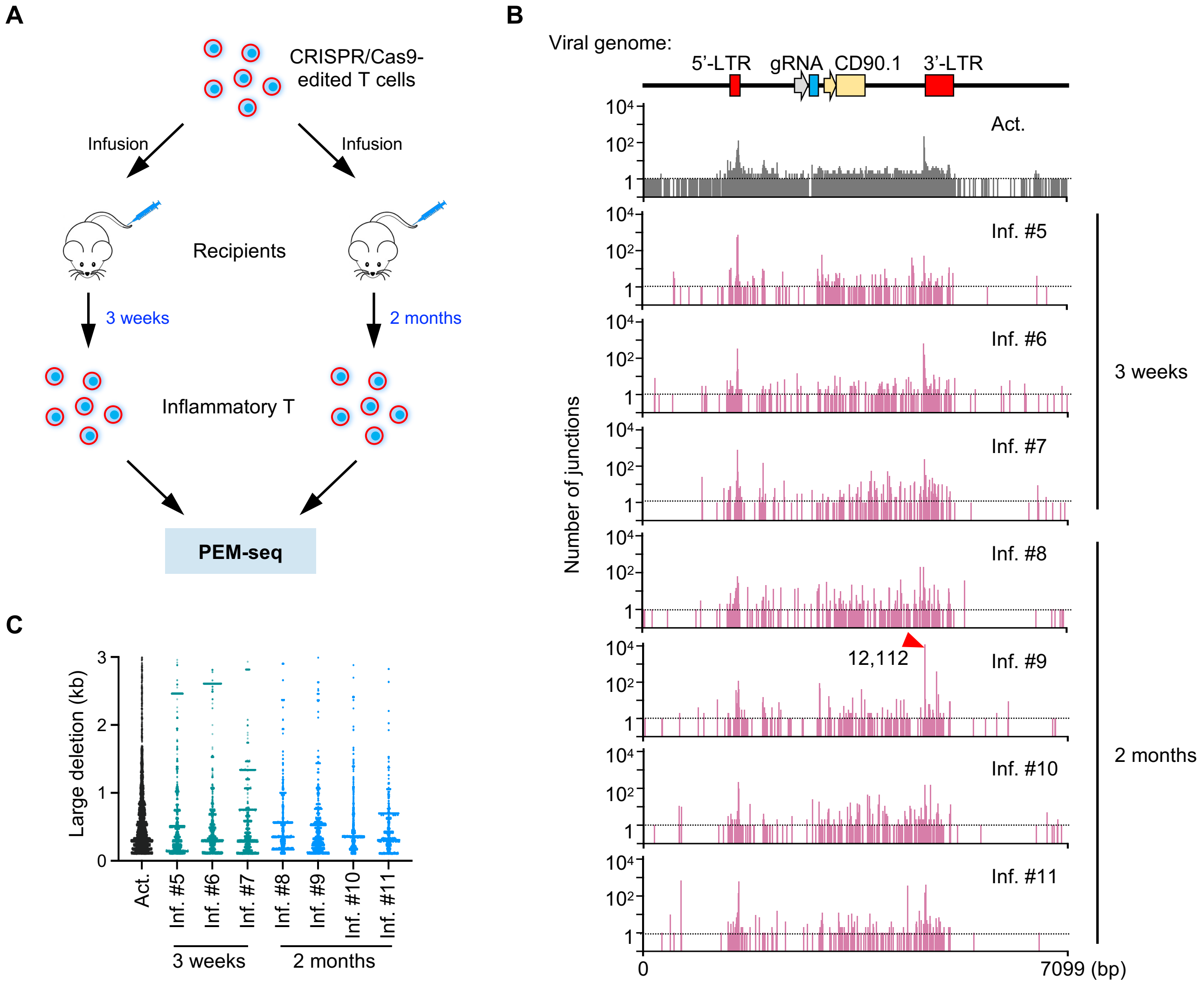

Fig. S6 Distribution of viral DNA insertions and large deletions in edited T cells post 2 months infusion.

A. Experimental procedures for tracing the *in vivo* progression of SVs in engineered T cells post 3-week or 2-month-infusion. CRISPR/Cas9 edited T cells are treated as described in Fig. 1**A** and then infused into *Rag1*$^{-/-}$ recipients with antigen stimulation. Three weeks or two months after infusion, the engineered donor-derived inflammatory T cells in the large intestine were sorted for PEM-seq analysis to comprehensively quantify each editing outcome.

B. Distribution of junctions in the viral genome with a 1-bp bin. The functional elements of the viral DNA are shown on the top. Red triangle indicated the viral integration with the highest number (12,112 after normalization) in Inf. #9.

C. Distribution of large deletions with a deletion length of 100-3000 bp per 1-bp bin. Each dot represents a unique junction.

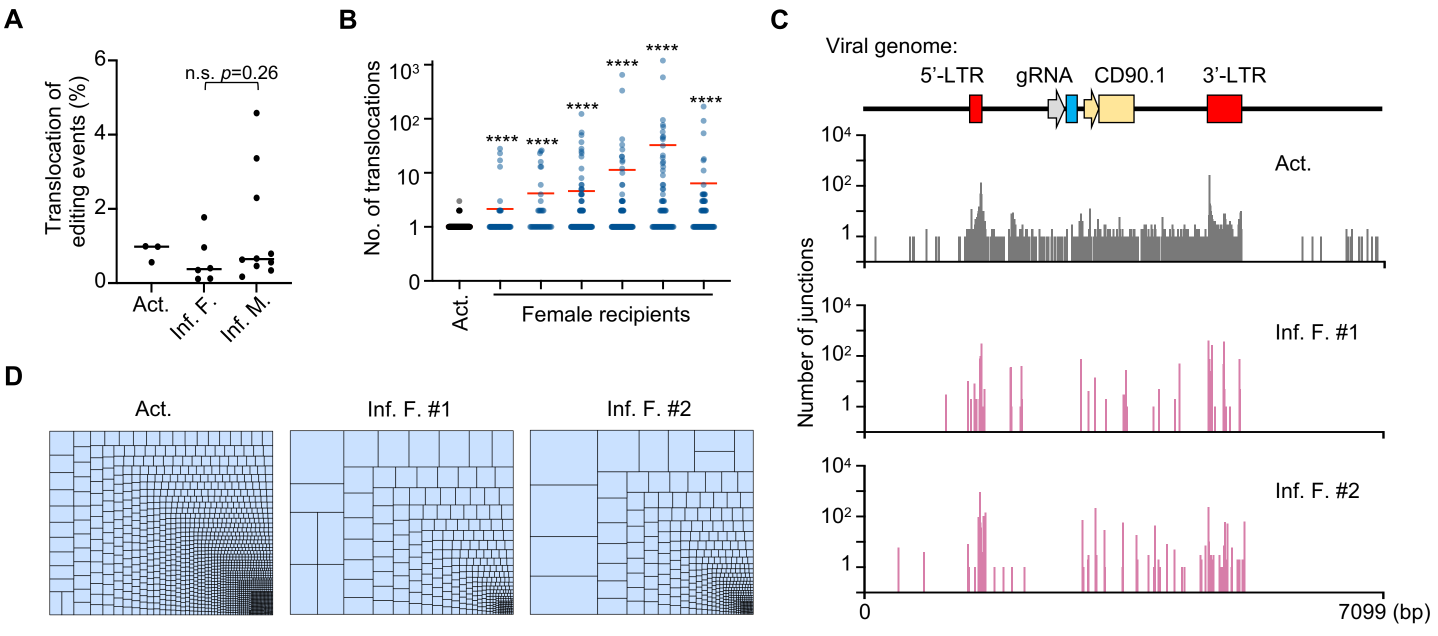

Fig. S7 Persistence and expansion of SVs in female recipients.

A. Percentages of chromosomal translocations in female (Inf. F.) or male (Inf. M.) recipients after infusion for 3 weeks. n.s., no significance; two-tailed *t*-test. Each dot represents a unique mouse.

B. Dot plot shows distribution of the number of translocations at each junction site with a 1-bp bin in female recipients. Each dot represents a unique translocation in one sample. Red lines show the median. ****, $p<0.0001$; two-tailed Mann–Whitney U test.

C. Distribution of junctions in the viral genome with a 1-bp bin. The functional elements of the viral DNA are shown on the top.

D. Tree map showing the proportion of junctions with a deletion length within 100-3000 bp. Each rectangle represents a unique large deletion with a 1-bp bin, and the area of the rectangle represents percentage of the indicated junction.
